# The Early Stages of *Pedaliodes poesia* (Hewitson, 1862) in Eastern Ecuador (Lepidoptera: Satyrinae: Pronophilina)

**DOI:** 10.1673/031.009.3801

**Published:** 2009-06-02

**Authors:** Harold F. Greeney, Tomasz W. Pyrcz, Philip J. DeVries, Lee A. Dyer

**Affiliations:** ^1^ Yanayacu Biological Station & Center for Creative Studies, Cosanga, Ecuador c/o 721 Foch y Amazonas, Quito, Ecuador; ^2^ Zoological Museum of the Jagiellonian University, lngardena 6, 30–060 Kraków, Poland; ^3^ University of New Orleans, Department of Biological Sciences, New Orleans, LA 70148; ^4^ Biology Department, University of Nevada; Reno, Nevada

**Keywords:** Andes, bamboo, *Chusquea*, cloud forest, larva, Poaceae, pupa

## Abstract

We describe the immature stages *Pedaliodes poesia* Hewitson, 1862 from northeastern Ecuador. *Chusquea scandens* (Poaceae, Bambusoidea) is the larval food plant. Eggs are laid singly or in pairs on the bottom side of host plant leaves. The duration of the egg, larval, and pupal stages, combined, is 99–107 days.

## Introduction

*Pedaliodes poesia* (Hewitson, 1862) (Figures 4–5) belongs to the subtribe Pronophilina, an Andean section of the subfamily Satyrinae (*sensu* Miller, 1968, with subsequent modifications, e.g., Lamas et al. 2004). It was originally described in the genus *Pronophila* Westwood. Butler (1867), however, erected the genus *Pedaliodes*, removing 25 species from *Pronophila*, and designating *P. poesia* as its type species. Nearly a hundred years later, Forster (1964) divided *Pedaliodes* — by this time containing 45 species — into several smaller groups based on male genitalic morphology and wing colour patterns. Forster's classification has been retained, with minor modifications, until the present time (Adams and Bernard 1981; Adams 1986; Pyrcz and Viloria 1999; Viloria 2007). Viloria (2007) characterised *Pedaliodes* as follows: butterflies of medium size, females slightly variable in wing pattern; males possessing androconial patches on FW discal area; both sexes with ocellar elements reduced or absent, when present, visible ventrally on post-discal area, most notably on HW. Compared to genera related to *Pedaliodes*, the male genitalia are characterised by: a robust and well developed uncus, generally as long as tegumen or slightly shorter; gnathi variable in length but always present; aedeagus generally thick and asymmetrically contorted; valvae generally with small dorsal processes; ampullar process present but variable (occasionally absent).

*Pedaliodes poesia* is fairly widespread, being found in central (Junín, Pasco, Huánuco) and northern (Amazonas, San Martín, Cajamarca) Peru on the eastern Andean slope, throughout the eastern slope of Ecuador, in all three Cordilleras of Colombia, including the Pacific slope of the Western Cordillera, and in the El Tamá range of south-western Venezuelan (Lamas et al. 2004). Similar to its congeners, *P. poesia* occurs within well-defined, midaltitudinal (1800–2600 m) bands (Adams 1985; Pyrcz and Wojtusiak 1999; Pyrcz 2004). However, this species is most abundant between 2000 and 2400 m; within this range *P. poesia* is one of the co-dominant species of the *Pedaliodes* assemblage (Pyrcz and Wojtusiak 1999). In Ecuador, *P. poesia* typically flies with *ca*. 20 congeners (Pyrcz. unpubl.).

The type locality of *P. poesia* was vaguely defined as “New Granada” (Hewitson, 1862), which at that time encompassed Colombia and large parts of Ecuador. The lectotype (Viloria 1998) [male, New Granada, Brit. Mus. Nat. His., type No. 3952; examined by Pyrcz], however, corresponds with individuals occurring in the eastern Cordillera in Colombia (Cundinamarca). Although no subspecies have been identified so far, populations of *P. poesia* differ consistently from one another in body size and wing pattern, especially the extent of white speckling on the ventral hindwing (HW), and the presence of red patches on the dorsal forewing (FW) (Adams 1986; Pyrcz 2004). Some of these species likely deserve subspecific status, in particular the Chocó and El Tamá populations, which show unique morphological characters (Pyrcz pers. obs). The population found at YBS does not differ noticeably from that at Cundinamarca. *Pedaliodes poesia* is closely related to several allopatric species: to *P. piletha* Hewitson of the Venezuelan Cordillera de la Costa; to *P. japhleta* Butlerin the Cordillera de Mérida (Colombia); to *P. suspiro* Adams and Bernard in the Sierra de Perijá (Colombia); and in the south to *P. hewitsoni* Staudinger, found in southeastern Peru (Cuzco, Puno) and northern Bolivia. All these taxa are morphologically and ecologically so closely-related, that they may eventually be considered a single species (Pyrcz pers. obs.). Sexual dimorphism in *P. poesia* is well developed, particularly when compared with other species of *Pedaliodes*. It is expressed mostly in the much lighter ventral HW pattern of the females, marked with wide, whitish or silvery patches and bands. Females are also slightly larger, and have somewhat more undulated outer margins of the HW. In some populations, females bear an orange or reddish dorsal FW patch, but this is never apparent in males. Sexual dimorphism in *P. poesia* and related species is so pronounced that it has previously led to the description of the two sexes as separate species. For example, *Pronophila phanaraea*, described by Hewitson (1868) is in fact a female of *P. poesia* from Ecuador (Thieme 1905). Similarly, *P. plautius* Grose-Smith is a female of *P. hewitsoni* (Lamas et al. 2004). Within *P. poesia*, there is also considerable individual wing pattern variation, mostly expressed in the lighter elements on the ventral surface of the HW.

Many species of *Pedaliodes* use bamboo species in the genus *Chusquea* (Poaceae) as their primary host plant (Adams 1986; DeVries 1987; Pyrcz and Wojtusiak 1999; Viloria and Heredia 2004). Other species, such as *Pedaliodes manis* C. and R. Felder, *P. plotina rapha* Pyrcz and Viloria, and *P. palaepolis* Hewitson, are associated with heavily disturbed areas. Larvae of these feed on secondgrowth grasses, such as *Festuca* and *Poa* (Pyrcz pers. obs). Despite the fact that over 270 species of *Pedaliodes* are distributed throughout the Andes (Viloria 2002), the ecology and morphology of immature stages is almost completely unknown,. To date, life histories have been well described for only two species: *P.phoenissa* (Hewitson) (Schultze 1929) and *P. zingara* Viloria and Heredia (Heredia and Viloria 2004). Here we supplement this knowledge with a description of the early stages of *P. poesia* from northeastern Ecuador.

## Materials and Methods

We carried out rearing and field investigations at the Yanayacu Biological Station and Center for Creative Studies (YBS: 00°35.949 S, 77°53.403 W), located in Napo Province, in the Andes of northeastern Ecuador. The study site is located approximately five kilometers west of the town of the town of Cosanga, adjacent to Cabañas San Isidro, and includes around 2000 hectares of primary cloud forest bordered by cattle pasture and other disturbed habitats [see Greeney et al. (2006) and Valencia (1995) for more complete descriptions of the study area]. We collected larvae at elevations ranging from 2000 to 2200 m, and reared them in glass jars at the ambient research lab, located at 2150 m.

We reared several larvae from 4 instar to eclosion, and two individuals from eggs (found in the field) to adults. We added fresh food plant as needed, removing frass and old leaves daily. We made larval measurements the day prior to molting.

## Results

### Egg (Figures 1 a-c)
n = 3; approx. 1.2 mm diameter; time to hatching > 11 days)

**Figure 1.**
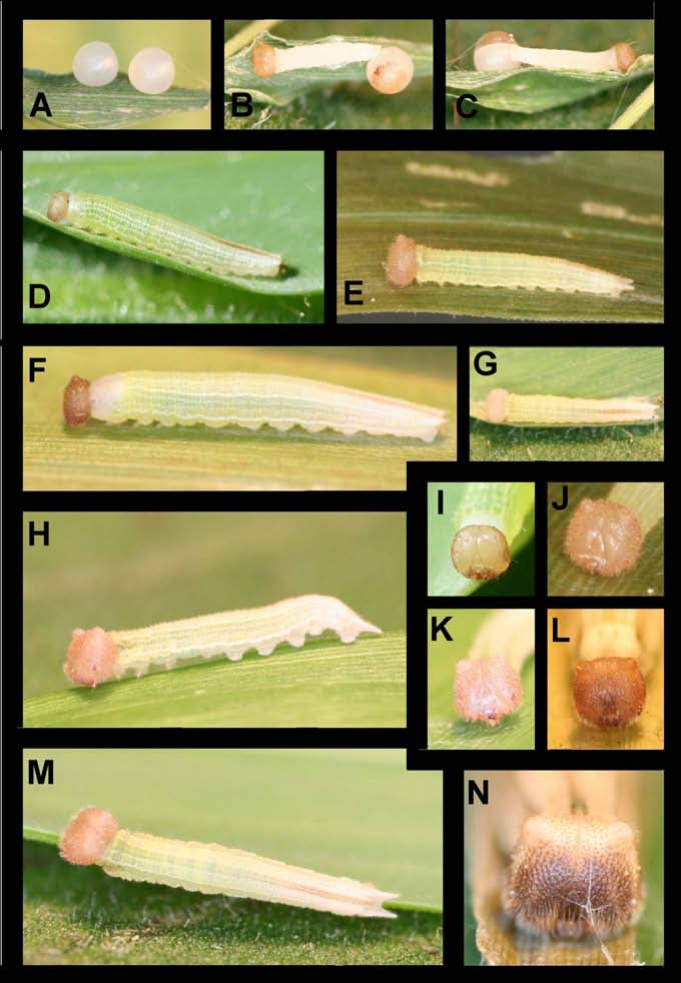
Immature stages of *Pedaliodes poesia* at YBS: a) freshly laid eggs; b-c) recently hatched larva and pre-hatching egg; d) pre-molt first instar; e) recently molted second instar; f) pre-molt second instar; g-h recently molted third instar); i) first instar; j) second instar; k) third instar; I) fourth instar; m) third instar; n) fifth instar.

Round, white, appearing smooth, but with minute, irregular, vertically oriented striations visible under dissecting scope (Figure 1a); laid singly (n =1) or in pairs (n = 1); prior to emergence, larval head capsules clearly visible below chorion (Figures 1b-c); upon emergence, larvae consume entire egg shell.

### First instar (Figures 1 c-d, i)
n = 2; body length = 2.5–6 mm; development time = 12–14 days

Head nearly round, caramel-colored, minutely reticulated with sparse, long pale setae; body at hatching white (Figures 1b-c), widest at Al, tapering gradually posteriorly and terminating in a pair of poorly developed caudal tails; soft pale setae laterally, most concentrated on A9–A10; prothoracic shield weakly sclerotized, clear and barely noticeable (Figure 1c); later in stadium (Figure 1d), larvae are greenish from host plant material visible in the gut; body round in cross-section and developing five pairs of indistinct longitudinal white stripes beginning on A4, darkening posteriorly, and terminating on A9; just prior to molting larvae develop a distinct red-brown middorsal stripe; caudal tails become more visible, but remain short.

### Second instar (Figures 1 e-f, h)
n = 2; body length = to 9 mm; development time = 8–10 days

Head roughly quadrate, caramel brown, finely pitted and reticulated with moderately dense, short, pale setae, epicranium bearing two short, rounded horns; body round in cross-section, similar in coloration to late first instar, except dorso-lateral and ventro-lateral white longitudinal stripes becoming more contrasting than others; body now bears minute granulations and sparse, short pale setae, caudal tails longer and more evident, and more widely separated.

### Third instar (Figures 1g-h, k, m)
n = 2; body length = to 12 mm; development time = 9 days

Head and body similar to second instar, but indistinctly square in cross-section; body coloration overall more yellowish; head horns and caudal tails slightly more pronounced.

### Fourth instar (Figures 11, 2a-c)
n = 4; body length = to 19.5 mm; development time = 11–12 days

**Figure 2.**
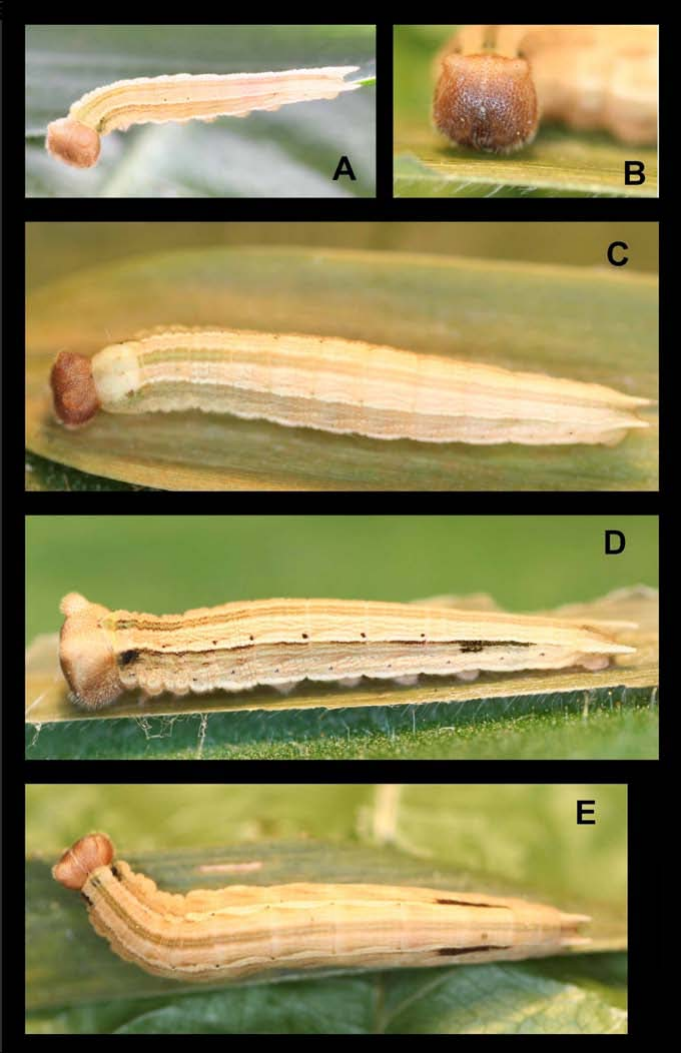
Immature stages of *Pedaliodes poesia* at YBS: a) recently molted fourth instar; b) fourth instar; c) pre-molt fourth instar; d) recently molted fifth instar; e) mature fifth instar.

Head similar to third instar, horns slightly more pronounced, light caramel-colored at molting but darkening to orange-brown as larva matures, darker anteriorly (Figure 2b); body similar in form to third instar, caudal tails longer, distinctly square in cross-section; general body coloration notably different from 3^rd^ instar, thoracic segments with distinct greenish highlights, darkening and extending to A3 later later in this stadium;, terminal segments, and A6, A7 also with greenish highlights; body ground color beige with thin, longitudinal, dorso-lateral and ventro-lateral white stripes; mature 4^th^ instar with a thick, mid-dorsal stripe running entire length of body, greenish anteriorly, fading to reddish centrally, then to greenish again posteriorly, entire body with hints of rose-colored blush; later in stadium developing pairs of indistinct, small, dark spots dorsolaterally at intersegmental sutures on A2 through A5.

### Fifth instar (Figures In, 2d-e)
n = 5; body length = to 23 mm; development time = 197–20 days, including pre-pupa

Head paler than in previous instars but with distinctly darker anterior portion, epicranial horns well defined but still short and rounded (Figure In); body trapezoidal in cross-section, narrower dorsally, widest around A3, ground color pale beige with fine, indistinct, wavy beige to pinkish patterning, mid-dorsal stripe well defined, greenish to A3 and from A9–10, reddish brown centrally, greenish portions lined subdorsally with dark brownblack; six pairs of small, distinct dark brown, dorsolateral spots from T3-A5; dorsolateral white stripe thickening into indistinct crescents on central abdominal segments and entire stripe to A9 subtended by distinct but irregular, thin, dark brown stripe, this stripe thicker, extending ventrally into square patches on T1 and A6; spiracles dark; late in stadium most pattern fading (see pre-pupal description) except for dark areas on T1 and A6–A7.

### Pre-pupa (Figures 2e, 3a-b)
n = 2; length = 23–25 mm; development time= 2–3 days

Similar to late fifth instar, but body almost entirely translucent white-pink to beige, silk pad white to beige.

### Pupa (Figures 3c-f, 4) n = 2; length = 13–14 mm; development time = 29–31 days

Overall form robust, roughly rectangular but with blunt edges except for indistinct, rounded ridges dorso-laterally on abdomen and head tapering slightly and squared off anteriorly; ground color pale beige with irregular brown flecking and patterning, patterning most distinct and extensive mid-dorsally and darkest in three triangular patches, two just dorsal of wing pads and one centrally on dorsal portion of head which extends posteriorly onto thorax; spiracles dark; cremaster continuous with abdomen, similar in color and only slightly sculptured; several days before eclosion, the entire pupa becomes dark brown (Figure 4).

## Discussion

Unfortunately, the paucity of published life history information on *Pedaliodes* leaves us with few species for comparison. The two published, complete life histories [*P. phoenissa* (Schultze 1929) and *P. zingara* (Heredia and Viloria 2004)], are similar in all life stages to *P. poesia*. Notably, third instars and pupae (the only stages illustrated by Schultze) of *P. phoenissa* appear nearly identical to those of *P. poesia*. In all three species, the later instars resemble dead, slightly moldy, rotting, or mossy plant parts. The early stages of *P. zjngara* show a different pattern from the other two species, but are similar in overall shape. Later instars of *P. zingam* are more strongly marked on the head and body. Pupae, however, vary among species in their shape. For example, the pupa of *P. poesia* is robust and rounded, while that of *P. zingam* is elongate and angular. The pupa of *P. zingara* bears lateral thoracic keels and cephalic projections, but that of *P. poesia* lacks distinct keels on the abdomen and thorax.

Apart from variation in pupal shape, the most striking difference between *P. poesia* and *P. zingara* is the light brown first instar head capsule of *P. poesia*, in contrast with the shining black head of first instar *P. zingara*. Also, while Heredia and Viloria (2004) report a mean life-cycle duration of about 80 days for *P. zingara*, our observations suggest that *P. poesia* may take as much as a month longer to develop from egg to adult. Schultze did not specify the complete life-cycle duration of *P. phoenissa*, but the period from third instar to imago lasts roughly 50 days, which indicates a development time similar to that of *P. poesia*. Heredia and Viloria (2004) suggest that the shorter life cycle of *P. zingara* (when compared to *Parapedaliodes parepa* and *P. phoenissa*; Schultze 1929, Pelz 1997) may be related to the lower altitudes (and presumably warmer temperatures) where it occurs (1900–2200 m).

**Figure 3.**
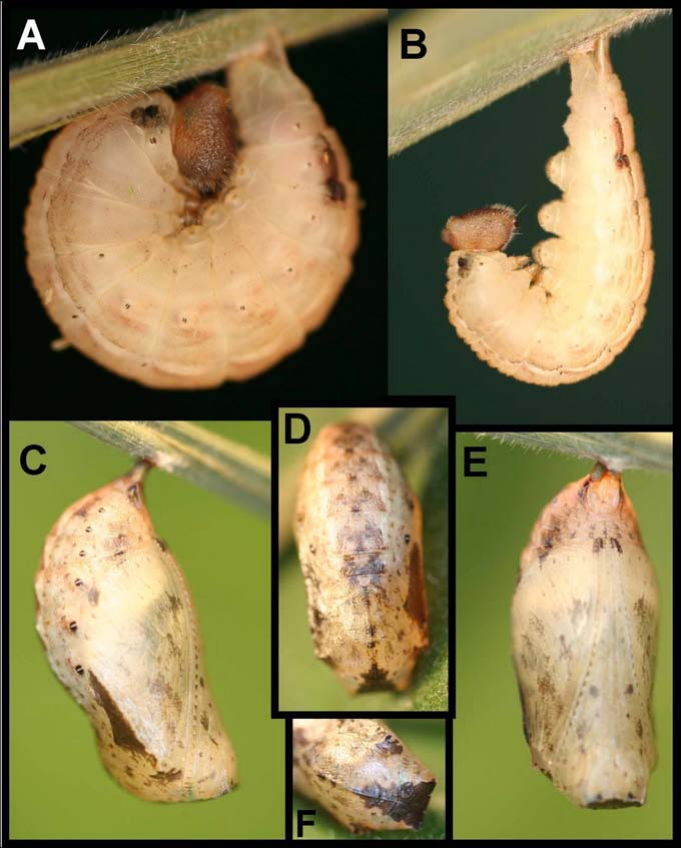
Immature stages of *Pedaliodes poesia* at YBS: a-b) pre-pupal larva; c-f) pupa.

We found few differences between larvae of *P. zingara* and *P. poesia* compared to those of *P.parepa* (Pelz 1997). It appears that larvae of *P. parepa* are rounder in cross-section, rather than flattened across the top, and are thus less trapezoidal in cross section than the two *Pedaliodes* spp. Similarly, the head scoli of *Parapedaliodes* appear to be somewhat more conical or pointed than the rounded, robust scoli of *Pedaliodes*. These caterpillars are similar in coloration, but *Parapedaliodes* exhibits a shining black head capsule like that of *P. zingara*. The strongly angular shape of *Parapedaliodes* pupae is similar to *P. zingara*. Comparing gross morphology of all three, it appears that the pupa of *P. zingara* is somewhat intermediate between *P. poesia* and *Parapedaliodes* in its “angularity.”

Like most Pronophilina, *P. poesia* larvae feed on *Chusquea*, a widespread bamboo integral to the Andean landscape. Nevertheless, our knowledge of pronophiline natural history is in its infancy. As the morphology and behavior of immature stages are known to provide important phylogenetic information in butterflies (e.g., Devries et al. 1985; Brown and Freitas 1994; Penz 1999; Freitas et al. 2002), we encourage the description of immatures for other pronophiline species, in an effort to make their larval characters available for phylogenetic analyses.

**Figure 4.**
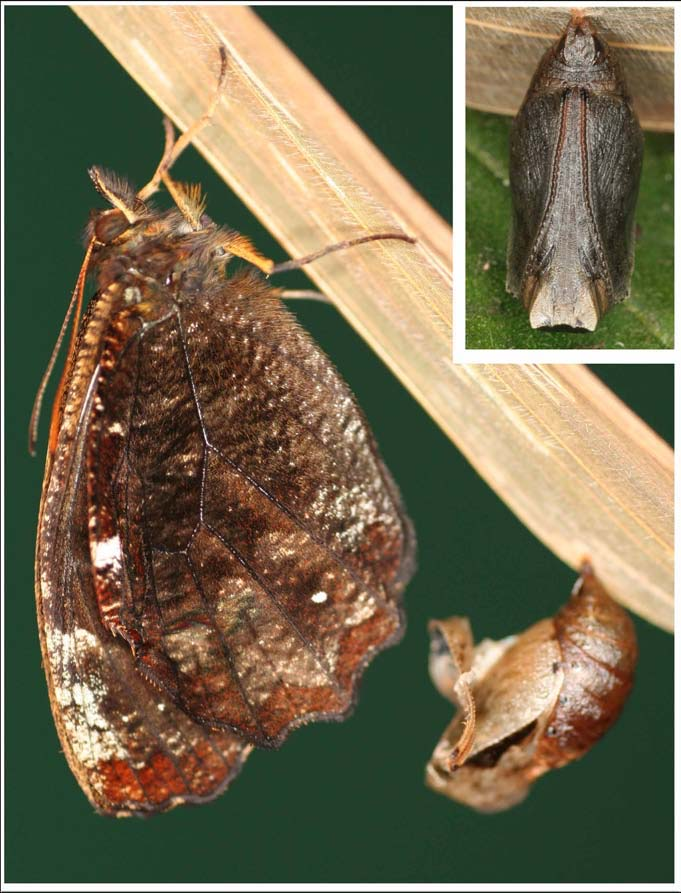
Adult *Pedaliodes poesia* emerging from its pupa at YBS. Inset shows the pupa just prior to hatching.

**Figure 5.**
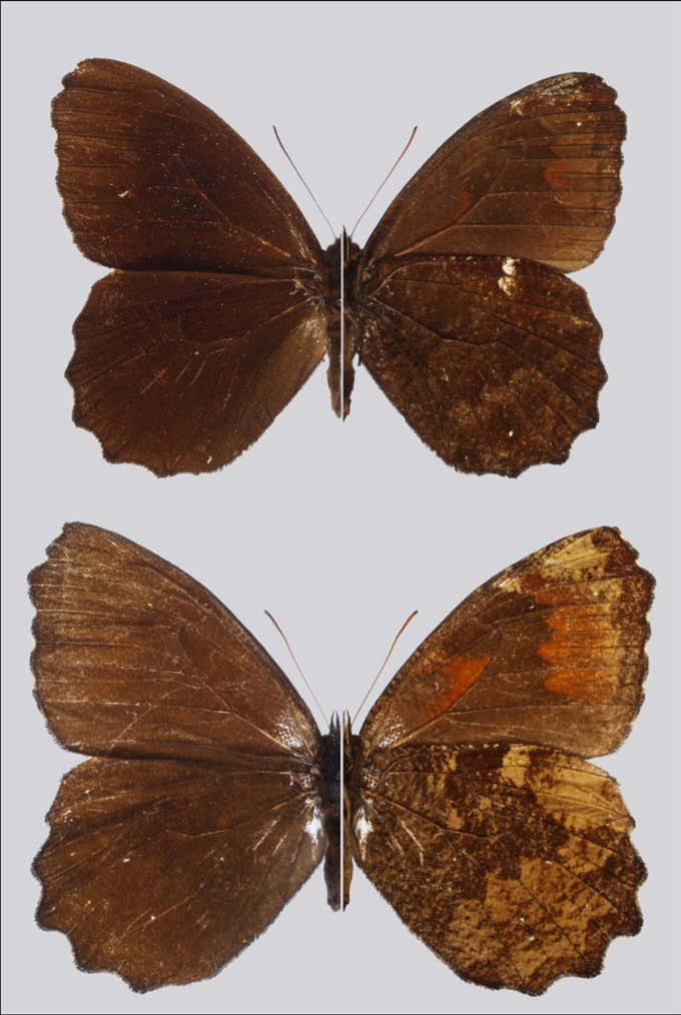
Adults of *Pedaliodes poesia*, dorsal surface on left and ventral surface on right. Upper specimen: ♂; Ecuador, Napo, Baeza- Tena, near Arrayan, 2000 m, 20 April 1998, A. Neild leg., MZUJ. Lower specimen: ♀; Ecuador, Tungurahua, Biscaya, 2100–2300 m, 06–07 May 1996, A. Jasi ski leg., Muzeum Zoologiczne Uniwersytetu Jagiellonskiego (Zoological Museum of the Jagiellonian University).

### Editor's note

Paper copies of this article will be deposited in the following libraries. Senckenberg Library, Frankfurt Germany; National Museum of Natural History, Paris, France; Field Museum of Natural History, Chicago, Illinois USA; the University of Wisconsin, Madison, USA; the University of Arizona, Tucson, Arizona USA; Smithsonian Institution Libraries, Washington D.C. USA; The Linnean Society, London, England.
